# Effects and Mechanisms of Cutting Upper Thoracic Sympathetic Trunk on Ventricular Rate in Ambulatory Canines with Persistent Atrial Fibrillation

**DOI:** 10.1155/2021/8869264

**Published:** 2021-02-02

**Authors:** Jie Cai, Min Tang, Hao Liu, Shiao Ding, Rongxin Lu, Wei Wang, Nan Ma, Ju Mei, Zhaolei Jiang

**Affiliations:** Department of Cardiothoracic Surgery, Xinhua Hospital, School of Medicine, Shanghai Jiaotong University, Shanghai 200092, China

## Abstract

**Objective:**

The purpose is to observe the effects and neural mechanism of cutting upper thoracic sympathetic trunk (TST) on the ventricular rate (VR) during persistent atrial fibrillation (AF).

**Methods:**

Twelve beagle dogs were halving to the control group and experimental group, 6 dogs for each group. Both groups were performed with left atrial rapid pacing (600 beats/min) to induce sustained AF. The experimental group underwent cutting upper TST  after a sustained AF model was established, while the control group received thoracotomy without cutting TST. Bilateral stellate ganglion (SG) and left atrial myocardium were harvested for tyrosine-hydroxylase (TH) immunohistochemical staining.

**Results:**

After cutting upper TST for 30 minutes, the average VR was 121.5 ± 8.7 bpm (95% CI, 114.8 to 128.0) in the experimental group, which was significantly slower than that of the control group (144.5 ± 4.2 bpm (95% CI, 141.5 to 148.0)) (*P* < 0.001). After cutting upper TST for 1 month, the average VR of the experimental group (106.5 ± 4.9 bpm (95% CI, 102.0 to 110.0)) was also significantly slower versus that of the control group (139.2 ± 5.6 bpm (95% CI, 135.0 to 143.8)) (*P* < 0.001). Compared with the control group, both left stellate ganglion (LSG) and right stellate ganglion (RSG) of the experimental group caused neural remodeling characterized by decreased ganglionic cell density and reduced TH staining. TH-positive component was significantly decreased in the left atrium of the experimental group compared with the control group.

**Conclusions:**

Cutting upper TST could reduce fast VR during persistent AF. Cutting upper TST induced bilateral SG neural remodeling and reduced sympathetic nerve density in the left atrium, which could contribute to the underlying mechanism of VR control during AF.

## 1. Introduction

Atrial fibrillation (AF) is the most common arrhythmia in clinical practice, which has high morbidity and mortality [1]. Both rhythm and rate controls are acceptable strategies in managing patients with AF [2]. Autonomic nerve activity has been associated with an increase in atrial arrhythmogenesis by acting as a trigger that can induce atrial tachyarrhymias such as atrial tachycardia (AT) and AF [3–5]. Increasing sympathetic nerve activity plays an important role in the occurrence and maintenance of AF [6–8]. Thoracic sympathetic trunk (TST) is the most important resource of cardiac sympathetic nerve, which has become a novel target for AF management [6–9].

Stellate ganglion (SG) is the most important component of upper TST [7, 9]. At present, few studies have achieved blocking TST by ablating or resecting SG to inhibit the induction of paroxysmal atrial tachycardia (PAT) or paroxysmal atrial fibrillation (PAF) [8, 10]. However, resecting the upper part of SG may cause Horner's syndrome; the effects of cutting TST by only resecting the lower part of the left stellate ganglion (LSG) on persistent AF and cardiac neural remodeling still remain uncertain. The purpose of this study is to observe the effects of cutting upper TST by only resecting the lower part of LSG on the ventricular rate (VR) during persistent AF in ambulatory canines. Also, this study observes the effects of cutting upper TST on the cardiac neural remodeling of the bilateral SG and left atrium in the canine model of persistent AF, which may be a possible mechanism of the antiarrhythmic effect of cutting upper TST.

## 2. Methods

The animal protocol was approved by the Institutional Animal Care and Use Committee in Xinhua Hospital, Shanghai Jiaotong University School of Medicine, and conformed to the Guide for Care and Use of Laboratory Animals (XHEC-F-2018-057). Twelve mature healthy male beagle dogs (Animal Laboratory Center, Xinhua Hospital, Shanghai Jiaotong University School of Medicine) weighing 15–25 kg were randomly halving to the control group and experimental group, 6 dogs for each group. The control group was merely performed with rapid left atrial pacing (600 beats/min) to induce persistent AF. The experimental group was disposed with rapid left atrial pacing (600 beats/min) and received cutting upper TST after persistent AF was documented.

### 2.1. Establishment of the Sustained AF Model

All canines of both groups were disposed with rapid left atrial pacing to construct a persistent AF model. Each canine was injected with Zoletil (10–15 mg/kg, intramuscular) to induce anesthesia and maintained with 2%∼4% isoflurane after endotracheal intubation and mechanical ventilation and then underwent thoracotomy through the fourth intercostal space on the left chest. Two epicardial pacing electrodes were stitched onto the left atrial appendage separately at a distance of 2 centimeters, which was connected to an Implantable Wireless Device of Electrocardiography (ECG) Acquisition and Stimulator (Ensen-ESST-79-5, Enshi Medical Technology (Shanghai) Co., Ltd.). Another two electrodes were stitched to the subcutaneous tissue of the left chest to record ECG signal. After pacing parameters were set appropriately (pacing model, continuously stimulating; voltage, 1500 mV; frequency, 10 Hz; pulse width, 1 ms), the implantable device was subcutaneously positioned on the left chest. Each canine received 3-day antibiotics after surgery with 0.5 g/d cefuroxime. After one week of postoperative recovery, rapid (600 bpm) left atrial pacing was then given continuously for one week. After one week, the stimulating model of the device was turned off to determine the presence of sustained AF (lasting >48 hours) [11, 12]. If the canine was not sustained AF, the atrial pacing continued for another week and the ECG was monitored weekly until sustained AF was documented.

### 2.2. Cutting Upper Thoracic Sympathetic Trunk and Recording VR

**Figure 1:**
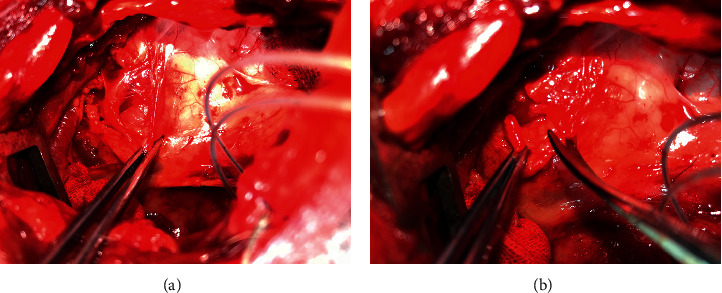
Cutting upper TST. (a) LSG (yellow arrow) was exposed on the top of the left thoracic cavity. (b) The lower part of LSG was resected (yellow arrow).

After the establishment of sustained AF, canines were continuously monitored for another 2 weeks. The canines of the experimental group then underwent the procedure of cutting upper TST by only resecting the lower part of LSG through the left third intercostal space, while the canines of the control group only received the thoracotomy without cutting upper TST. After the left third intercostal thoracotomy, honeycomb and adipose tissue were separated around the base of the 7^th^ cervical vertebra and the first rib, and LSG was exposed on the top of the left thoracic cavity **(**Figure 1(a)**)**; then the lower part of LSG was resected **(**Figure 1(b)).

ECG was recorded, respectively, at different time points (for the experiment group, before anesthesia, 30 minutes after anesthesia and before cutting TST, 30 minutes after cutting TST; for the control group, before anesthesia, 30 minutes after anesthesia and before thoracotomy, 30 minutes after thoracotomy). Each canine received 3-day antibiotics with 0.5 g/d cefuroxime after the second surgery. ECG was recorded again after one month of recovery. ECG was used for VR analysis. Then, the dog was euthanized.

### 2.3. Immunohistochemistry Studies

Bilateral SG tissue and left atrial myocardial tissue of all dogs were obtained and fixed in 4% formalin for 45 mins, followed by storage in 70% alcohol for tyrosine-hydroxylase (TH) immunohistochemical staining using an anti-TH antibody (22941, Immunostar, USA). The tissues were paraffin-embedded and cut into 5 *μ*m thick sections routinely. All slides were examined manually under a DP72 microscope (Olympus, Tokyo, Japan). A blinded observer pictured randomly select 200X fields with the highest ganglion cell density. The mean number of ganglion cells and the mean percentage of TH-negative ganglion cells were calculated. The densities of TH-positive nerves within the left atrial myocardial tissue were determined with Image J software.

### 2.4. Data Analysis

The data were reported as mean ± standard deviation (SD) and 95% confidence interval (CI). All data were tested for normality using the D'Agostino and Pearson normality test. Paired *t*-test was performed to compare the differences at different experimental time points in the same group. Independent *t*-test was performed to compare the differences between the experimental group and control group. For the data with nonnormality, Wilcoxon rank-sum test was used to compare the data between groups. A *P* value of ≤0.05 was considered statistically significant.

## 3. Results

### 3.1. Sustained AF Model Establishment

After left atrial rapid pacing for 3∼6 weeks, a stable sustained AF model was successfully developed in all canines. There was no significant difference in AF inducing duration between the experimental group (4.2 ± 0.8 weeks (95% CI, 3.6 to 4.8)) and the control group (4.5 ± 1.0 weeks (95% CI, 3.7 to 5.3)) (*P*=0.541, *t* = −0.632).

### 3.2. Effects of Cutting Upper TST on VR during AF

Table 1 shows the effects of cutting upper TST on VR during AF at different time points in both the experimental group and the control group. After the establishment of sustained AF, average VR during AF was 157.3 ± 7.9 bpm (95% CI, 151.0 to 163.7) in the experimental group and 154.2 ± 5.8 bpm (95% CI, 149.8 to 158.8) in the control group (*P*=0.450, *t* = 0.787) before anesthesia in ambulatory dogs (AF baseline). After anesthesia and before cutting upper TST, there was no significant difference on average VR between the experiment group (143.7 ± 5.2 bpm (95% CI, 139.8 to 148.0)) and control group (146.0 ± 3.0 bpm (95% CI, 143.6 to 148.4)) (*P*=0.362, *t* = −0.954). After cutting upper TST for 30 minutes, the average VR was 121.5 ± 8.7 bpm (95% CI, 114.8 to 128.0) in the experimental group, which was significantly slower than that of the control group (144.5 ± 4.2 bpm (95% CI, 141.5 to 148.0)) (*P* < 0.001, *t* = −5.829). After cutting upper TST for 1 month, the average VR of the experimental group (106.5 ± 4.9 bpm (95% CI, 102.0 to 110.0)) was significantly slower versus that of the control group (139.2 ± 5.6 bpm (95% CI, 135.0 to 143.8)) (*P* < 0.001, *t* = −10.763).

### 3.3. Effects of Cutting Upper TST on Neural Remodeling of LSG

**Figure 2:**
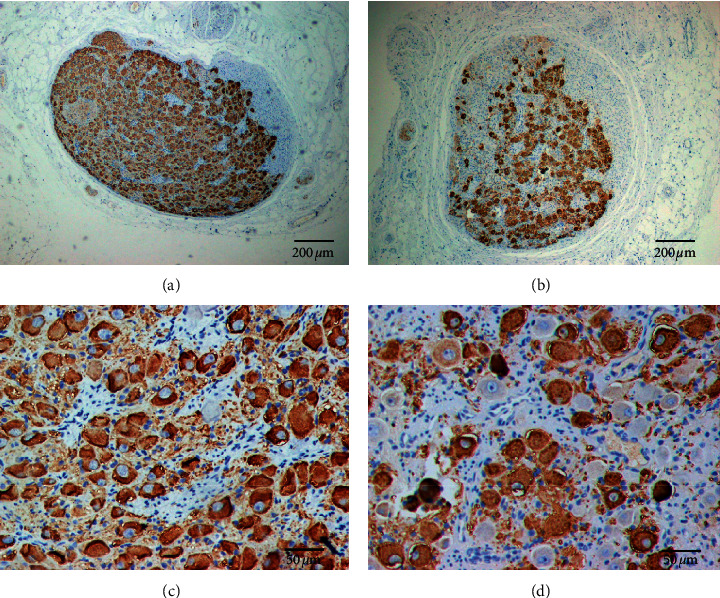
TH staining of LSG in both control group and experimental group. (a) The LSG of the control group seen at low magnification. (b) The LSG of the experimental group seen at low magnification. Compared with that of the control group, TH staining of LSG was weaker in the experimental group. (c) The LSG of the control group seen at high magnification. TH staining showed TH-negative ganglion cells (red arrows) and TH-positive ganglion cells (brown color) in LSG. (d) The LSG of the experimental group seen at high magnification. Compared with that of the control group, TH staining showed that the number of ganglionic cells was significantly decreased, but the quantity of TH-negative ganglion cells was significantly increased in the LSG of the experimental group. Effects of cutting upper TST on the neural remodeling of right SG (RSG).

LSG tissues were successfully harvested for analyses in all canines.  Figure 2 shows an example of neural remodeling in LSG of the control group and experimental group. Compared with that of the control group, neural remodeling characterized by decrease ganglionic cell density and reduced TH staining were visible under low power field in all LSG studied after cutting upper TST for 1 month in the experimental group. Compared with that of the control group (76.3 ± 0.8, 95% CI: 74.8–77.9), the mean ganglionic cell number was significantly decreased in the LSG of the experimental group (58.7 ± 2.3, 95% CI: 54.1–63.2) (*P* < 0.001, *t* = 7.160). The mean percentage of TH-negative ganglionic cells in LSG of the experimental group (28.2% ± 3.2%, 95% CI: 21.9%–34.5%) was significantly higher than that in LSG of the control group (6.3% ± 0.6%, 95% CI: 5.1%–7.5%) (*P* < 0.001, *t* = 6.711).

**Figure 3:**
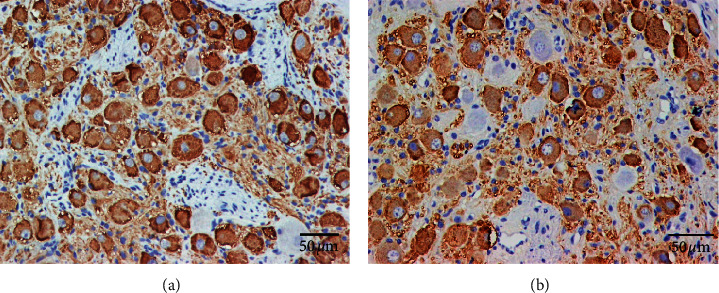
TH staining of RSG in both control group (a) and experimental group (b). (a) The RSG of the control group seen at high magnification. TH staining showed TH-negative ganglion cells (red arrows) and TH-positive ganglion cells (brown color) in RSG. (b) The RSG of the experimental group seen at high magnification. Compared with that of the control group, TH staining showed that the number of ganglionic cells was significantly decreased, but the quantity of TH-negative ganglion cells was significantly increased in the RSG of the experimental group.

Right SG (RSG) of all canines was successfully obtained for analyses.  Figure 3 shows an example of neural remodeling in RSG of the control group and experimental group. TH staining showed TH-negative ganglion cells (red arrows) and TH-positive ganglion cells (brown color). Compared with the control group (73.5 ± 2.3, 95% CI: 69.0–78.0), the mean ganglionic cell quantity of RSG was significantly decreased in the experimental group (63.2 ± 1.6, 95% CI: 60.1–66.2) (*P*=0.004, *t* = 3.713). Besides, the mean percentage of TH-negative ganglionic cells in RSG of the experimental group (12.8% ± 0.9%, 95% CI: 11.1%–14.6%) was significantly higher than that of the control group (5.4% ± 0.8%, 95% CI: 3.8%–7.1%) (*P* < 0.001, *t* = 6.074).

### 3.4. Effects of Cutting Upper TST on Neural Remodeling of the Left Atrium

**Figure 4:**
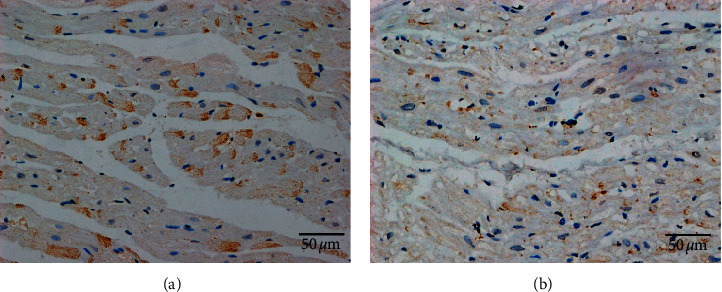
TH staining of the left atrium in both control group (a) and experimental group (b). (a) The left atrium of the control group seen at high magnification. (b) The left atrium of the experimental group seen at high magnification. TH staining showed that the TH-positive component (red arrows) was significantly decreased in the left atrium of the experimental group compared with that of the control group.

Left atrial myocardial tissues of all canines were successfully obtained for analyses.  Figure 4 shows a typical example of neural remodeling in the left atrium between the two groups. Compared with that of the control group (4.1‰ ± 0.4‰, 95% CI: 3.3‰–4.9‰), the mean TH-positive area ratio in the left atrial myocardium was significantly decreased in the experimental group (2.2‰ ± 0.2‰, 95% CI: 1.8‰–2.6‰) (*P*=0.002, *t* = 4.041).

## 4. Discussion

This study demonstrated the following: (1) cutting upper TST is efficient in controlling fast VR during AF in ambulatory dogs with pacing induced sustained AF and (2) cutting upper TST could cause cardiac neural remodeling and reduce the sympathetic density in bilateral SG in canines with sustained AF, which is beneficial to inhibit cardiac sympathetic activity and control fast VR during persistent AF.

### 4.1. The Relationship between Cutting Upper TST and VR during AF

Persistent AF can induce cardiac remodeling including electrical remodeling, which may have fast VR and decrease cardiac function. Both rhythm and rate controls are acceptable strategies in managing patients with persistent AF [2, 13]. The autonomic nervous system plays an important role in the occurrence and maintenance of AF [3–5]. In the last few decades, alternative strategies of VR control including vagal nerve stimulation (VNS) were developed [11, 12, 14, 15]. Upper TST is the most important resource of the cardiac sympathetic nerve, which has become a novel target for AF management [6–9].

SG is the most important component of upper TST, which is a sympathetic ganglion formed by the fusion of the inferior cervical ganglion and the first thoracic ganglion [7, 9]. Previous studies have shown that SG nerve activity (SGNA) was related to VR acceleration and spontaneous cardiac arrhythmias, and decreasing SGNA may be useful in inhibiting cardiac arrhythmias and reducing fast VR [16–18]. Shen et al. demonstrated that chronic left low-level VNS could effectively suppress left SGNA and reduce the incidence of PAT in ambulatory dogs [14]. In addition, Chinda et al. also found that intermittent VNS could lead to reduced SGNA and VR control during persistent AF in ambulatory dogs [12]. However, the effects of cutting upper TST by only resecting the lower part of LSG on persistent AF and cardiac neural remodeling are uncertain. In this study, we observed the effects of cutting upper TST by only resecting the lower part of LSG on VR. Compared with that of the control group, the average VR was significantly slower at both 30 minutes and one month after cutting upper TST in the experimental group. Our results demonstrated that cutting upper TST was efficacious in controlling fast VR and tachyarrhythmias during persistent AF in ambulatory dogs.

### 4.2. Cutting Upper TST Causes Bilateral SG Neural Remodeling in Canines with Sustained AF

Sympathetic tone is important in cardiac arrhythmogenesis. Several studies have shown that SGNA was related to VR acceleration and cardiac arrhythmias, and decreasing SGNA may be helpful in inhibiting cardiac arrhythmias [16–18]. Previous studies have shown that the VNS could suppress SGNA, inhibit the occurrence of PAF, or decrease VR during AF by inducing SG neural remodeling. These studies showed that VNS caused neural remodeling of the SG with a decreased density of TH-positive ganglion cells and more TH-negative ganglion cells [12, 14].

In this study, the mean ganglionic cell number was significantly decreased in both LSG and RSG of the experimental group compared with that of the control group. The mean percentage of TH-negative ganglionic cells in LSG and RSG of the experimental group was significantly higher than that in LSG and RSG of the control group. The results demonstrated that cutting upper TST induced bilateral SG neural remodeling, which was one of the possible mechanisms of VR control of cutting upper TST. However, the neural remodeling of LSG appeared to be more significant than that of RSG.

### 4.3. Cutting Upper TST Causes Cardiac Neural Remodeling in Canines with Sustained AF

The cardiac nervous system includes the intrinsic cardiac nervous system (ICNS) which is composed of nerve structures that are inside the heart and the extrinsic cardiac nervous system (ECNS) which is composed of nerve structures that are outside of the heart. Both the ECNS and ICNS are known to be related to an increase in atrial arrhythmogenesis or fast VR [6, 19, 20]. Choi et al. have shown that there was a significant temporal relationship between extrinsic cardiac nerve activity (ECNA; including stellate ganglion nerve activity and vagal nerve activity) and intrinsic nerve activity (ICNA; including epicardial ganglionated plexi nerve activity and ligament of Marshall nerve activity), indicating that there is a communication between ECNS and ICNS [19]. Stimulating the ECNS via VNS has been shown to be effective in suppressing the occurrence of AF and reducing VR during persistent AF [12, 14, 15].

In this study, we not only found that cutting upper TST could cause both LSG and RSG neural remodeling, but also found that cutting upper TST could reduce the sympathetic nerve density in the left atrial myocardial tissue. Compared with that of the control group, TH-positive component was significantly decreased in the left atrium of the experimental group. The results indicate that cutting upper TST could reduce cardiac sympathetic outflow, which may be another possible mechanism of VR control of cutting upper TST.

## 5. Study Limitations

The present study has two limitations. Firstly, we did not evaluate the SG function by directly recording SGNA. Secondly, we only observed the effect of cutting upper left TST. The effect of cutting upper right TST or bilateral TST should be studied in the future.

## 6. Conclusions

Cutting upper TST could reduce fast VR during AF in ambulatory dogs with pacing induced sustained AF. Cutting upper TST induced bilateral SG neural remodeling and reduced the sympathetic nerve density in the left atrium, which could contribute to the underlying mechanism of VR control during AF.

## Figures and Tables

**Table 1:** The effects of cutting upper TST on VR during AF.

Parameter	Experimental group (bpm)	Control group (bpm)	*t* value	*P* value
Before (AF baseline)	157.3 ± 7.9	154.2 ± 5.8	0.787	0.450
After anesthesia and before cutting upper TST	143.7 ± 5.2^*∗*^	146.0 ± 3.0^*∗*^	−0.954	0.362
After cutting upper TST for 30 minutes	121.5 ± 8.7^*∗*^	144.5 ± 4.2	−5.829	<0.001
After cutting upper TST for 1 month	106.5 ± 4.9^*∗*^	139.2 ± 5.6^*∗*^	−10.763	<0.001

^*∗*^ *P* < 0.05 versus AF baseline.

## Data Availability

The data are stored in the Department of Cardiothoracic Surgery, Xinhua Hospital, School of Medicine, Shanghai Jiaotong University, Shanghai (200092), China.
